# Signaling Pathways in Exosomes Biogenesis, Secretion and Fate

**DOI:** 10.3390/genes4020152

**Published:** 2013-03-28

**Authors:** Lorena Urbanelli, Alessandro Magini, Sandra Buratta, Alessandro Brozzi, Krizia Sagini, Alice Polchi, Brunella Tancini, Carla Emiliani

**Affiliations:** 1 Department of Experimental Medicine and Biochemical Sciences, University of Perugia, Via del Giochetto, 06123 Perugia, Italy; E-Mails: urbanel@unipg.it (L.U.); alessandro.magini@libero.it (A.M); alessandro.brozzi@gmail.com (A.B.); krma@iol.it (K.S.); alicepolchi@virgilio.it (A.P.); brunellatancini@virgilio.it (B.T.); 2 Department of Internal Medicine, Section of Biochemistry, University of Perugia, Via del Giochetto, 06123 Perugia, Italy; E-Mail: sandra.buratta@unipg.it; 3 Centro di Eccellenza sui Materiali Innovatovi Nanostrutturati (CEMIN), University of Perugia, Via Elce di Sotto 8, 06123, Perugia, Italy

**Keywords:** : exosome, extracellular vesicles, microvesicles, endosome, lysosome, multivesicular bodies, signaling, cell-to-cell communication, Wnt signaling, biomarkers

## Abstract

Exosomes are small extracellular vesicles (30–100 nm) derived from the endosomal system, which have raised considerable interest in the last decade. Several studies have shown that they mediate cell-to-cell communication in a variety of biological processes. Thus, in addition to cell-to-cell direct interaction or secretion of active molecules, they are now considered another class of signal mediators. Exosomes can be secreted by several cell types and retrieved in many body fluids, such as blood, urine, saliva and cerebrospinal fluid. In addition to proteins and lipids, they also contain nucleic acids, namely mRNA and miRNA. These features have prompted extensive research to exploit them as a source of biomarkers for several pathologies, such as cancer and neurodegenerative disorders. In this context, exosomes also appear attractive as gene delivery vehicles. Furthermore, exosome immunomodulatory and regenerative properties are also encouraging their application for further therapeutic purposes. Nevertheless, several issues remain to be addressed: exosome biogenesis and secretion mechanisms have not been clearly understood, and physiological functions, as well as pathological roles, are far from being satisfactorily elucidated.

## 1. Introduction

Two decades of research have provided evidence that many kinds of cells shed small vesicles, which play a key role in intercellular communication. Three main types of vesicles have been described so far: microvesicles (100 nm to 1 μm in diameter), which directly bud from the plasma membrane, apoptotic blebs (50–500 nm), which are released by cells undergoing apoptosis, and exosomes (30–100 nm), released via exocytosis from multivesicular bodies (MVBs) of the late endosome [1,2]. Whereas microvesicles and apoptotic blebs appear more heterogeneous in shape, exosomes show a characteristic cup-shaped or well delimited round morphology, as observed by electron microscopy [3]. 

Exosomes are usually purified by serial steps of centrifugation and ultracentrifugation [4] and recovered at 100,000 × g as pellets [5]. For further purification, ultracentrifugation on linear sucrose gradient (2–0.25 M sucrose) is required [4], with exosomes floating to a density ranging from 1.13 to 1.19 g/mL [5]. Some steps of serial centrifugation can be skipped through filtration on 0.22 μm pore filters [3]. Recently, 50–100 nm vesicles enriched in classical markers of exosomes, displaying similar density on sucrose gradient and size by electron microscopy, have been described to bud from the plasma membrane [6]. These vesicles cannot be distinguished from endosome-derived exosomes and have further complicated their definition. In addition, this finding clearly showed that the simple purification by differential ultracentrifugation is not sufficient to qualify vesicles as exosomes, and a combination of quantitative protein composition, morphological (electron microscopy) and physical (floatation on sucrose gradients) criteria should be always used to identify exosomes among other extracellular vesicles [7].

Following the initial description in reticulocytes [8], several cell types have been shown to release exosomes in extracellular medium *in vitro*, such as hematopoietic cells (B-, T-, dendritic and mast cells, platelets), epithelial cells, neural cells (oligodendrocytes, neurons, microglia and Schwann cells), adipocytes, fibroblasts, stem cells and many types of tumor cells. Of relevance, exosomes are found *in vivo* in several biological fluids, such as saliva, urine, plasma, seminal fluid, amniotic liquid, ascites, bronchoalveolar lavage fluid, synovial fluid, breast milk and cerebrospinal fluid (CSF) [9].

## 2. Exosome Content

Apart from their morphological and physical properties, exosomes are usually identified on the base of their unique protein and lipid composition: as our knowledge on proteins specifically associated with exosomes will improve, this would probably allow for an easier procedure of exosome purification by affinity. Because of their endosomal origin, exosomes contain proteins involved in membrane transport and fusion (e.g., Rab GTPases, annexins, flotillins), MVB biogenesis (e.g., Alix and tumor susceptibility gene 101 or Tsg101) or protein families mostly associated with lipid microdomains, such as integrins and tetraspanins (e.g., CD63, CD9, CD81 and CD82) [10]. According to proteomic studies, exosome protein composition is different from membrane vesicles released by apoptotic cells and membrane shedding microvesicles [11]. Exosome protein content includes both conserved proteins, identified in almost all exosomes despite their origin, and cell-type-specific proteins. Among conserved proteins, the heat shock cognate 70 kDa protein (hsc70) and the tetraspanin, CD63, are the most frequently identified [12]. Moreover, other frequently found proteins are linked to the cytoskeleton (β-actin, myosin, cofilin and tubulins) or to metabolism (e.g., glyceraldehyde 3-phosphate dehydrogenase (GADPH, ENO1). In agreement with their role as antigen presenting vesicles, many exosomes contain major histocompatibility complex (MHC) class I and II molecules. Of interest, exosomes contain also proteins involved in cell signaling pathways, like β-catenin, Wnt5B or the Notch ligand, Delta-like 4, and mediators of intercellular cell signaling, like interleukin-1β, TNF-α or TGF-β. However, the influence of these mediators on target cell signaling pathways remains largely to be elucidated [13].

As for proteins, the lipid composition of exosomes is distinct from that of the cell of origin, but is anyway characteristic of the cell type. In addition, exosomes show some common lipid features independently of their origin. Lipid composition analysis has been performed with exosomes derived from hematopoietic cells [14], oligodendrocytes [15] and melanoma cells [16]. Internal membranes of MVBs are enriched in lipids, such as lysobisphosphatidic acid (LBPA) [17]. LBPA plays an important role in exosome biogenesis [18]: it interacts strongly with Alix, a protein also involved in exosome biogenesis, and can induce internal budding of small vesicles from large liposomes when added to a lipid mixture mimicking the composition of the MVB membrane [19]. Moreover, a decrease of the LBPA level reduces the number of vesicles in MVBs [20]. Another characteristic of exosomes is their enrichment in lipids usually associated with lipid rafts, such as cholesterol, sphingolipids, ceramide and glycerophospholipids with long and saturated fatty-acyl chains [15,21]. Furthermore, exosomes also contain many lipids which are signaling mediators, such as prostaglandins, the enzyme responsible for the release of the prostaglandin synthesis intermediate arachidonic acid (phospholipase A2), a phospholipid scramblase and phospholipases C and D, both involved in the release of signaling mediators from membrane phospholipids [22].

A major breakthrough in the field has been the discovery that exosomes secreted by mast cells contain nucleic acids, such as mRNA and miRNA [23]. As mRNAs were also showed to be translated in target cells, this was the first evidence suggesting cell-to-cell communication by exosome-mediated transfer of genetic information. Exosome-transferred miRNAs were also suggested to be functional in target cells, as miRNAs from T-cell exosomes caused inhibition of target genes in dendritic cells (DCs) [24], and miRNAs contained in exosomes from Epstein–Barr virus infected B-cells affected the expression of target genes in monocytes [25]. Not all mRNAs present in a cell are included in exosomes, so there is a specific targeting of mRNA into exosomes, whose mechanisms are unknown. Moreover, it is not clear whether a set of mRNAs is targeted to all exosomes despite their cell of origin, in addition to cell-type-specific mRNAs. On the miRNAs side, it is still unclear whether specific miRNA sequences, rather than the whole set of intracellular miRNAs, are targeted to exosomes. Recently, it was also reported that neural cells [26] and myoblasts [27] release exosomes carrying mitochondrial DNA (mtDNA). The role of exosomal mtDNA is unknown, although it has been suggested that it could reach the cytosol of the target cells and be imported into the mitochondria. 

## 3. Exosome Biogenesis

**Figure 1:**
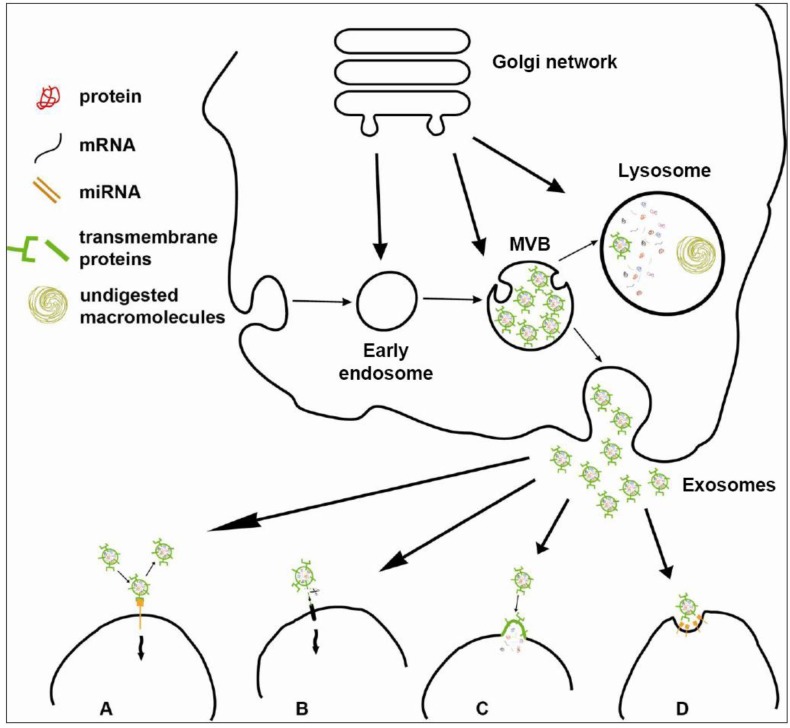
The biogenesis of exosomes and their interaction with target cells. Exosomes originate from multivesicular bodies (MVBs), late endosome-derived cell compartments, which bud off parts of their limiting membrane into their lumen, forming intraluminal vesicles. MVBs can either fuse with the lysosome for degradation or with the plasma membrane to release exosomes into the extracellular space. Exosomes mediate cell-to-cell communication through different mechanisms of interaction with target cells. **(A)** Exosomes activate intracellular signaling by ligand-receptor interaction, without internalization. **(B)** Extracellular proteases cleave exosomal membrane proteins, releasing soluble ligands that bind to target receptors on the cell surface. **(C)** Target cells take up exosomes by membrane fusion, so exosomes release their content into the cytoplasm. **(D)** Target cells internalize exosomes by endocytic mechanisms (phagocytosis, macropinocytosis or receptor-mediated endocytosis)**.**

Exosomes originate in the intraluminal vesicles (ILVs) of MVBs, which fuse with the cell surface in an exocytic manner [10]: as late endosomes bud off parts of their limiting membrane into the lumen of late endosomes, vesicles are formed inside the lumen (ILVs) originating this particular type of late endosome, which accumulates hundreds of ILVs and is termed MVB (Figure 1). Initially, it was thought that MVBs could just fuse with lysosomes to degrade their intraluminal content. This process was important in order to remove transmembrane proteins, as well as excessive membranes [28,29]. As a matter of fact, the degradation of transmembrane proteins is relevant for the downregulation of activated cell surface receptors, and abnormalities in this process have been implicated in cancer development [30]. Later it was demonstrated that in reticulocytes undergoing maturation into red blood cells, MVBs can also fuse with the plasma membrane to release their ILVs into the extracellular space, and the term “exosomes” was proposed to define these extracellularly released intra-endosomal vesicles [8].

### 3.1. Biogenesis of MVBs: The Role of the Endosomal Sorting Complex Required for Transport (ESCRT) Machinery

The process of MVBs formation is coordinated by the endosomal sorting complex required for transport (ESCRT). This complex consists of four soluble multiprotein complexes, called ESCRT-0, ESCRT-I, ESCRT-II and ESCRT-III, and it is usually recruited to the cytosolic side of the endosomal membrane for the sorting of selected protein to ILVs. This process is known to require ubiquitination of the cytosolic tail of endocytosed receptors [31]. Tsg101, which belongs to the ESCRT-I complex, is recruited to form a complex binding the ubiquitinated cargo proteins and activating the ESCRT-II complex, which, in turn, initiates the oligomerization and the formation of the ESCRT-III complex. This complex participates in the sequestration of MVB proteins and recruits a deubiquitinating enzyme, which removes the ubiquitin tag from the cargo proteins prior to sorting them into the ILVs. Finally, the ESCRT-III complex is disassembled by an ATPase [32,33].

While the ESCRT proteins are clearly required for the targeting of membrane proteins for lysosomal degradation, the function of the ESCRT machinery in the formation of ILVs secreted as exosomes is less clear. As a matter of fact, the identification by proteomic analysis of members of the ESCRT complex, such as Alix and Tsg101, in DC exosomes strongly supports an ESCRT-dependent exosome biogenesis [34]. In addition, the involvement of an ESCRT-0 member in the secretion of DC exosomes was also reported [35]. On the other hand, exosomal targeting of MHC class II molecules in activated DCs does not require ubiquitination [36]. Moreover, in oligodendroglial cells, no role for Tsg101 or Alix was found in proteolipid protein (PLP) sorting into exosomes [15]. In melanocytes, the sequestration of the premelanosomal protein, Pmel17, in ILVs also appears to be independent from ESCRT function [37]. Altogether, these studies suggest that subpopulations of MVBs using different machinery for their biogenesis could exist in different cell types or, alternatively, co-exist in the same cell type [10].

### 3.2. Biogenesis of Exosomes: ESCRT-Independent Pathways

As mentioned, several studies have provided evidence that some exosomal proteins are released in an ESCRT-independent manner. Different alternative pathways have been proposed to be involved. Trajkovic and colleagues have shown that Tsg101 or Alix have no roles in the exosomal sorting of PLP, but the secretion of PLP containing exosomes requires ceramide [15]. Ceramide is a lipid with a cone-shaped structure, possibly facilitating membrane invagination of ILVs. A role for this lipid has been also suggested by studies demonstrating an involvement of sphingomyelinases (enzymes generating ceramide from sphingomyelin) in the biogenesis of exosomes, namely of acid sphingomyelinase in the release of vesicles from glial cells [38] and of neutral sphingomyelinase 2 in the secretion of miRNA-containing vesicles [39].

Other investigations have suggested that higher order oligomerization, *i.e.* the oligomerization of oligomers, may play a role in exosome biogenesis. This process was shown to be at the basis of CD43 exosomal sorting in Jurkat T-cells [40]. A similar mechanism has also been proposed for the transferrin receptor in reticulocytes [41] and the MHC class II complex in lymphocytes [42]. In both studies, the oligomerization of oligomers induced by antibody crosslinking was shown to enhance the secretion of investigated proteins into exosomes. 

The biogenesis of MVBs also correlates with the presence of detergent-resistant domains in exosomal membranes, in which members of the tetraspanin protein family are also co-localized. As a matter of fact, it was reported that the sorting of MHC class II into DC exosomes is partially dependent on its incorporation into tetraspanin CD9-containing lipid microdomains [36].

## 4. Regulation of Exosome Secretion

Exosome secretion is regulated by MVB fusion with the plasma membrane. Mechanisms underlying MVB fusion with the plasma membrane are not well known, but some studies have recently shed light in this field. Intracellular calcium changes in mast cells, human erythroleukemia cells [43], cultured cortical neurons [44] and oligodendrocytes [45] have been demonstrated to induce exosome release. The cell depolarization induced by K^+^ also increased the secretion of neuronal exosomes [44]. Furthermore, it has been demonstrated that in cultured cortical neurons, the exosomal release is modulated by glutaminergic activity [46]. Cell activation by crosslinking of receptors, such as IgE in mast cells [47,48] or CD3 in T-cells, triggers the release of exosomes [49]. In macrophages and DCs, the P2X purine receptor 7 regulates the ATP-mediated release of exosomes [50].

Intracellular trafficking and fusion of compartments often involve small GTPases of the Rab family. In human erythroleukemia cells the process involves the small GTPase Rab11 [51], and in T-cells the citron kinase, a rhoA effector [52], and Rab27 [53]. It was also shown that Rab27A and Rab27B play complementary roles in spontaneous secretion of MHC class II-bearing exosomes [53]. In addition to Rab11 and Rab27, Rab35 is involved in the secretion of PLP-enriched exosomes by oligodendroglial cells [54]. However, it is not clear whether different Rabs act at different steps of exosome secretion or are differently used by specific cell types. There is also evidence of the involvement of lipids in the regulation of exosome secretion. The lipid mediator phosphatidic acid, originating either from the activity of diacylglycerol kinase or phospholipase D, influences exosome secretion [55,56,57]. 

The final step of exosome secretion requires the fusion of MVBs with the plasma membrane. This process possibly involves a specific combination of Soluble N-ethylmaleimide-sensitive factor attachment protein receptors (SNAREs): vesicular SNAREs (v-SNAREs), localized on MVBs, interact with target SNAREs (t-SNAREs), localized on the intracellular side of the plasma membrane, to form a membrane-bridging SNARE complex, responsible for membrane fusion [58]. As a matter of fact, Fader and colleagues demonstrate that in a erythroleukemia cell line, the v-SNARE complex, named TI-VAMP/VAMP7, participates in the fusion between MVBs and the plasma membrane [59]. 

Many studies have shown that the secretion of exosomes is enhanced in cells undergoing stress conditions. This response depends on p53 activation, which regulates the transcription of tumor suppression-activated pathway 6 (TSAP6) gene, implicated in exosome biogenesis [60,61]. The p53 response to stress includes not only the production of secreted protein mediators, but also of exosomes, which can communicate with neighboring cells to influence their behavior. For example, exosomes secreted in a TSAP6-mediated manner are able to trigger the release of histamine from immune cells [62].

## 5. Exosomes as Mediators of Cell-to-Cell Communication

### 5.1. Exosome Interaction with Target Cells

Initially, exosomes were hypothesized to be an important system to dispose garbage for cells with poor lysosomal degradative capacity. In the following years, it became evident that exosomes are relevant for a variety of physiological processes, but even if many aspects of their *in vivo* function remain to be elucidated, they are now considered important for cell-to-cell communication, and many signaling pathways are probably influenced by exosomes. A few mechanisms for exosome-mediated cell-to-cell communication have been proposed (Figure 1): (i) exosome membrane proteins could interact with their receptors on target cells, thus activating intracellular signaling; (ii) exosome membrane proteins could be cleaved by proteases, and soluble fragments could act as soluble ligands binding to cell surface receptors; and (iii) exosomes could be internalized by target cells, releasing their content to activate downstream events in recipient cells [12].

About exosome interaction with membrane receptors on target cells (Figure 1A), many proteins have been described to function by this mechanism. Exosomes derived from mature DCs carry on their surface the intercellular adhesion molecule 1 (ICAM1) and were captured by ICAM1 binding to the lymphocyte function-associated antigen 1 receptor localized on the surface of antigen-presenting cells (APCs) [63] or activated T-cells [64]. A decrease of exosome uptake by DCs was observed during the simultaneous inhibition of α_v_ (CD51) and β_3_ (CD61) integrins, of CD11a and its ligand CD54, or by blockade of CD9 and CD81 tetraspanins by antibodies [65]. In the same study, an implication of lactadherin, which binds to α_v_β_3_ and α_v_β_5_ integrins, was established [65]. CD91, a receptor for many heat shock proteins, was also implicated in the interaction of exosomes derived from mast cells with T-cells, as an anti-CD91 treatment decreased exosome effects [47]. Because of an enrichment of phosphatidylserine (PS) in the outer layer of exosomes, multiple PS binding proteins on target cells could be susceptible to bind exosomes, and the PS receptor TIM-4 was actually described to be involved in this process [66].

The second mechanism hypothesized for exosome-target cell interaction was based on soluble ligands produced by proteolytic cleavage of exosomal membrane proteins (Figure 1B). Despite the fact that proteolysis of transmembrane proteins generally occurs at the cell surface, proteolytic processing could also occur either inside the cells, within MVBs or in released exosomes. Indeed, the L1 neural adhesion molecule and CD44 undergo proteolytic cleavage in MVBs by ADAM10 (A disintegrin and metalloproteinase 10), and the soluble ligands of L1 and CD44 are directly released from the cell via MVB exocytosis or, alternatively, full-length transmembrane forms are released in exosomes and then undergo further ADAM10-mediated cleavage in the extracellular space [67]. A similar mechanism of exosome-mediated release has been reported for full-length transmembrane CD46 in ovarian adenocarcinoma cell lines [68] and tumor necrosis factor receptor 1 (TNFR1) in vascular endothelial cells [69].

The third mechanism hypothesized for exosome-dependent cell-to-cell communication is internalization. Evidence has been reported for exosome fusion with recipient cells, in particular, when the transfer of genetic materials was investigated [23,70] (Figure 1C). However, fusion with the plasma membrane could be limited to acidic pH conditions, such as those found inside a tumor [16], as exosome and plasma membranes display the same fluidity at pH 5, but not at neutral pH, which makes membranes more rigid and less capable to fuse [22]. Alternatively, target cells could internalize exosomes by endocytic mechanisms. As a matter of fact, exosomes were described to be selectively transferred from oligodendrocytes to microglia by macropinocytosis [71], while in hematopoietic cell lines, they were shown to enter cells via phagocytosis and not by internalization pathways involving caveolae clathrin-coated vesicles or macropinocytosis [66] (Figure 1D). 

### 5.2. Biological Functions Regulated by Exosomes

Exosomes are secreted by hematopoietic, epithelial, neural, cancer and stem cells, so they are likely to be involved in many physiological and pathological processes. In the immune system, exosomes have been demonstrated to play an important role in the regulation of signals, mediating both adaptive and innate immune responses. In particular, there are several lines of evidence that exosomes are involved in spreading antigens or MHC–peptide complexes. As a matter of fact, studies on exosome-mediated activation of T-cells have shown that exosomal MHC-peptide complexes can directly bind to their cognate T-cell receptor [4,42,64,72], or alternatively, exosomes can be captured and processed by APCs and, thus activate T-cells [64,73]. Furthermore, the activating effect of exosomes appears to be dependent on the cell type, as exosomes from mature DCs were more efficient at inducing T-cell activation than those from immature DCs [72,74,75,76].

Exosomes present many types of antigens: exosomes from pathogen-infected cells contain pathogenic antigens, which can be associated with MHC molecules for presentation to T-cells after exosome capture by DCs [77,78]. Moreover, exosomes secreted from tumor cells represent a source of tumor antigens for capture and presentation by DCs, which induce efficient antitumoral immune responses [79,80,81]. However, the current literature reports the contradictory effects of tumor-derived exosomes, and whether exosomes preferably mediate antitumoral or immunosuppressive responses is unknown. As a matter of fact, several studies are in favor of the immunoppressive effect of exosomes, which can decrease proliferation of T-lymphocytes [82,83,84,85] and natural killer cells [86,87] or promote the differentiation of immunosuppressive cells, such as regulatory T-cells [88] or myeloid cells [87,89]. Interestingly, exosomes bearing immunosuppressive molecules seem to be relevant in the mother’s tolerance to the fetus, as placenta-derived vesicles from women delivering at term showed higher Fas ligand-mediated T-cell inhibiting properties [90].

Beyond immunomodulatory functions, exosomes have also been associated with additional tumor promoting activities. As a matter of fact, tumor exosomes display angiogenic properties [91] and hypoxic tumor cells enhance angiogenesis by exosome secretion [92]. Moreover, exosomes have also been implicated in the activation of relevant oncogenic pathways by secretion of epidermal growth factor receptor (EGFR) signaling ligands, such as amphiregulin, which is displayed on the surface of exosomes in a signaling-competent orientation [93]. The presence on the surface of exosomes of active ligands for receptors such as EGFR, which is overexpressed in many cancers, also suggests the possible involvement of exosomes in providing a favorable environment to circulating tumor cells for the development of metastasis [94]. The nucleic acid content of exosomes also appears to be important for tumor promoting properties: mRNA from glioblastoma exosomes can be translated into angiogenic proteins in endothelial cells [70] and colorectal cancer cell-derived microvesicles are enriched in mRNAs, promoting proliferation of endothelial cells [95]. The invasive properties of tumors were also affected by exosomes, as vesicles secreted from tumor-associated macrophages were reported to transport miRNA into breast cancer cells promoting cancer invasiveness [96].

A few studies also indicate an important role of the exosome pathway in retroviral spreading. HIV-1 particles captured by DCs are internalized into MVB-like compartments [97]; then, they exploit the exosomal pathway to move to T-cells by exocytosis, without *de novo* infection [98,99]. Such a system facilitates virus survival and propagation, as the internalized virus can escape lysosomal degradation, and upon fusion of the MVB with the plasma membrane, released viral particles can infect other cells without being recognized as “foreign agents”.

In the nervous system, exosomes have been shown to be secreted from many cell types, such as neurons, Schwann cells, oligodendrocytes and microglia. Exosomes isolated from a specific cell type were shown to affect other neural cell types; thus, it seems reliable that their main function could be to ensure communication within the central nervous system [44,45,46]. Neuronal MVBs are predominantly distributed within the somatodendritic compartment, where they are 50-times more abundant than in the axon [100]; thus, exosome release could occur from dendrites and modulate synaptic transmission and plasticity. The notion that exosome release is dependent on synaptic activity is supported by the finding that dendritic MVBs increase in electrically stimulated neurons [101].

As exosomes represent a mechanism of information transfer among neurons, a possible drawback could be the transmission of pathological proteins, such as the scrapie form of the prion protein (PrP^sc^), amyloid precursor protein (APP) fragments, phosphorylated tau or alpha-synuclein. As a matter of fact, it has been demonstrated that PrP^sc^, which is responsible of Creutzfeldt-Jacob disease, is secreted in exosomes [102]. In addition, it remains infective and can induce prion disease in mice [103]. Alpha-synuclein, whose aggregation is responsible for Parkinson’s disease, has been also described to be secreted in neuronal exosomes [104,105]. Alzheimer’s disease is characterized by the extracellular accumulation of insoluble amyloid fibrils of APP in the brain, and multiple lines of evidence indicate that amyloid peptides are released with exosomes [106,107,108,109].

Exosomes appear to be secreted also by stem cells, where they have been identified as paracrine mediators [110]. This finding has prompted extensive investigations to assess their regenerative properties. Exosomes from mesenchymal stem cells were injected into models of ischemia/reperfusion injury and reduced the infarct size [111]. In another study, exosomes secreted from endothelial cells and captured from smooth muscle cells were shown to reduce atherosclerotic lesion formation by a miR-143/145-mediated mechanism [112].

## 6. Exosomes as Regulators of Intracellular Signals: The Canonical Wnt/β-catenin Pathway

Proteomic studies have shown that exosomes contain proteins involved in cellular signaling pathways. The influence of these proteins on target cell signaling pathways is far from being completely elucidated, but recent reports have shed light in this field. In particular, Wnt signaling has attracted attention, because it plays critical roles during embryo development and tissue regeneration, as well as in cell proliferation disorders, namely cancer. However, mechanisms by which Wnt proteins are released and travel to target cells are mostly unknown. As a matter of fact, the palmitoylated Wnt proteins are membrane-bound and, thus, unlikely to be released as soluble proteins into the extracellular space. Recently, a trans-synaptic transduction of Wnt signaling through the release of exosome-like vesicular structures has been reported in *Drosophila* [113,114]. These findings have suggested the presence of a novel vesicular mechanism of trans-synaptic communication. In addition, Wnt proteins were shown to be directly secreted in exosomes, not only during *Drosophila* development, but also in human cells, and exosomes carrying Wnt proteins on their surface were reported to activate Wnt signaling in target cells [115].

Cytosolic β-catenin is the principal mediator of canonical Wnt signaling in target cells. The presence of β-catenin in exosomes was initially revealed by proteomic investigations [9]. Recently, it was reported that the CD9 and CD82 tetraspanins regulate Wnt signaling, reducing the intracellular pool of β-catenin. Interestingly, this reduction was due to the increase of exosome associated transport of β-catenin outside the cell [116]. This finding has suggested that exosomal packaging and release of cytosolic proteins could represent a new way to downregulate the activity of intracellular signaling pathways.

## 7. Conclusions

In the last decade, we have assisted an explosion of interest in the field of extracellular vesicles and, namely, of exosomes. These appear to be an additional and fundamental mechanism of cell-to-cell communication in many tissues and processes, but their physiological and pathological implications are still distant from being elucidated. The *in vivo* mechanisms of action of exosomes are not clear: we do not know how far they spread from the site of secretion and how many of them are secreted and captured by target cells. As a matter of fact, most studies were based on the *in vitro* administration of a large amount of purified exosomes, a condition that could be very different from what really happens *in vivo*, where these vesicles are continuously secreted and captured by surrounding tissues, and cells are exposed to exosomes from several sources at the same time. As a matter of fact, recently, it has been demonstrated that exosomes derived from *in vitro* passaged tumor cells show different properties with respect to cells directly isolated from the tumor mass [117]. Nevertheless, exosomes have become an extensively investigated source of biomarkers, potentially measurable in easily accessible body fluids. Furthermore, many clinical applications are emerging. In addition to their use as tumor vaccines [118], they are also promising gene delivery vehicles and tools for regenerative medicine, as they are able to cross the blood-brain barrier [119]. The accurate understanding of pathways involved in exosome biogenesis and secretion or in exosome-mediated cell-to cell communication will be of fundamental importance to address basic, as well as applicative issues.
